# Type 2 immune profile and impaired SMAD3-mediated repair in refractory stasis dermatitis responsive to IL-4Rα blockade

**DOI:** 10.1016/j.jdin.2026.03.015

**Published:** 2026-04-19

**Authors:** Chen Shen, Tingyu Liu, Lu Li, Guoqun Yu, Wen-Hung Chung, Juan Tao

**Affiliations:** a Department of Dermatology, Union Hospital, Tongji Medical College, Huazhong University of Science and Technology, Wuhan, China; b Hubei Engineering Research Center of Skin Disease Theranostics and Health, Huazhong University of Science and Technology, Wuhan, China; c Department of Dermatology, General Hospital of Central Theater Command of Chinese People’s Liberation Army, Wuhan, China; d Department of Dermatology, Chang Gung Memorial Hospital, Linkou, Taipei and Keelung, Taiwan; e Chang Gung Immunology Consortium, Chang Gung Memorial Hospital and Chang Gung University, Taoyuan, Taiwan; f Department of Dermatology, Xiamen Chang Gung Hospital, Xiamen, China; g College of Medicine, Chang Gung University, Taoyuan, Taiwan; h Immune-Oncology Center of Excellence, Chang Gung Memorial Hospital, Linkou, Taiwan; i Whole-Genome Research Core Laboratory of Human Diseases, Chang Gung Memorial Hospital, Keelung, Taiwan; j Genomic Medicine Core Laboratory, Chang Gung Memorial Hospital, Linkou, Taiwan

**Keywords:** atopic dermatitis, blood vessel, dupilumab, stasis dermatitis, tissue repair, type 2 inflammation

To the Editor:

Stasis dermatitis (SD) is a chronic inflammatory skin disease caused by venous insufficiency, leading to persistent inflammation and tissue injury, which may be complicated by eczematous appearance and chronic ulcers in severe cases. Conventional therapies such as topical or systemic corticosteroids, antibiotics, compression therapy, and even venous surgery often fail in refractory cases. Unlike atopic dermatitis (AD), ulcerative lesions are uncommon but characteristic in SD, reflecting distinct pathogenic mechanisms. Interestingly, while dupilumab has previously been reported to improve eczema-like SD lesions,^1^ our findings reveal that IL-4Rα blockade may contribute to the healing of ulceration, suggesting broader therapeutic potential in this condition.

We retrospectively reviewed 4 patients with long-standing, treatment-resistant SD, all presenting with severe pruritus and most undergoing persistent ulceration. The study was reviewed and approved by the Ethics Committee of Union Hospital, Tongji Medical College, Huazhong University of Science and Technology. Diagnosis was based on clinical features and duplex ultrasonography, with other causes of leg ulcers carefully excluded, including allergic contact dermatitis and AD (Supplementary Table I, available via Mendeley at https://doi.org/10.17632/hgr545cz2f.1). All patients had failed multiple standard therapies. Following dupilumab, all patients showed rapid responses, with visible improvement within 2 to 6 weeks and sustained remission during 17.5-30 months of follow up. Treatment led to marked reduction in erythema, ulcer size, and pain, with a mean venous clinical severity score (VCSS) decrease of 11.75 points during the biologic treatment (Fig 1, *A*; Table I).

**Fig 1:**
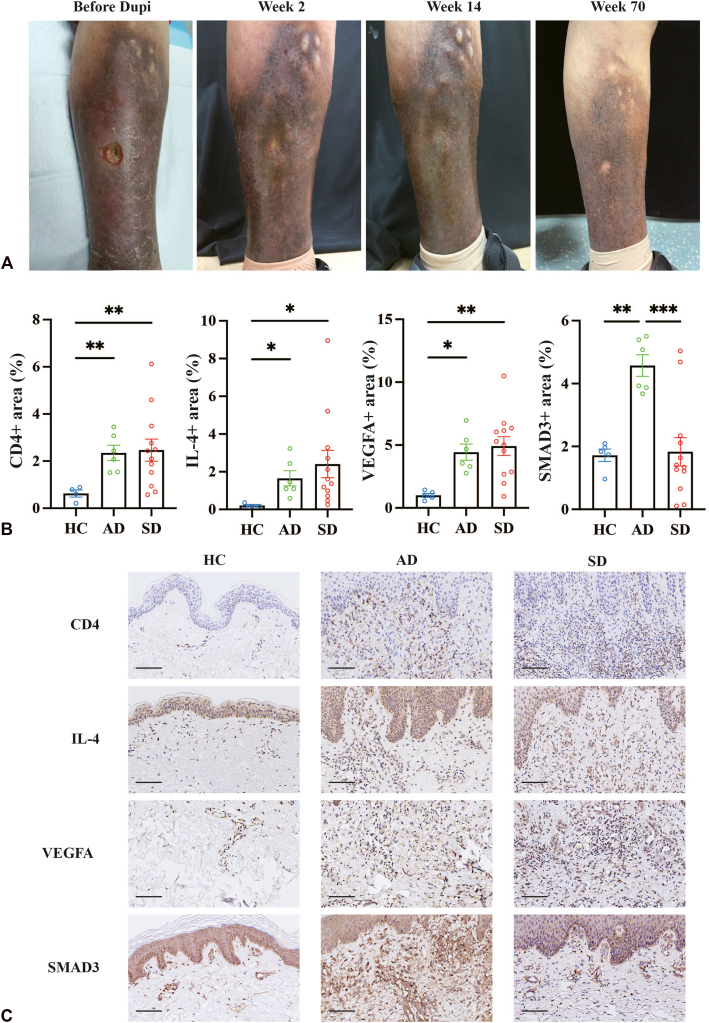
Clinical responses and molecular changes to dupilumab therapy in refractory patients with stasis dermatitis. A representative patient (Pt_1) presented with varicose veins, edematous erythema, hard lump and ulcers in the lower limbs. Following dupilumab treatment, her pruritus and pain have significantly improved and the ulcer achieved completely healing **(A)**. Relative expression levels of CD4, IL-4, VEGFA and SMAD3 in the dermis in SD, AD, and HCs. Data are presented as mean ± SEM. Statistical significance was determined using one-way analysis of variance followed by Tukey’s test or Brown-Forsythe analysis of variance followed by Dunnett T3 test for multiple comparisons. ∗*P* < .05, ∗∗*P* < .01, and ∗∗∗*P* < .001 **(B)**. Representative immunohistochemical images of CD4, IL-4, VEGFA and SMAD3 staining in skin lesions from the 3 groups. Scale bar = 100 μm **(C)**. *SD*, Stasis dermatitis; *AD*, atopic dermatitis; *HC*, healthy control.

**Table I:** Clinical characteristics and treatment outcomes of patients with SD treated with dupilumab

Patient	Age (y)/Sex	Duration (y)	Previous therapy∗	Disease history	Lesion location	Clinical features (bef)	Response to dupilumab (bef/aft)	Follow up (mo)
1	69/F	5	AH, TC, TA, SA, VVS	No ACD, no atopic feature	Lower legs and ankles	• Erythema, cellulitis, hard lump, itching • 2 cm ulcer, pain	Ulcer area: ∼2,300† → 0 (−100%) VCSS:23→8 (−65%)	17.5
2	59/M	5	AH, TC, SG, TA, DT	No ACD, no atopic feature	Predominantly lower legs; mild erythema on hands	• Lichenification, pigmentation, itching • Mild erosion, pain	Ulcer area: ∼1,800 → 0 (−100%) VCSS:7→2 (−71%)	20
3	55/F	4	AH, TC	No ACD, no atopic feature	Lower legs and ankles	• Erythematous patches, itching • Mild erosion and ulcer, pain	Ulcer area: ∼1,200 → 0 (−100%) VCSS:15→2 (−87%)	30
4	72/M	35	AH, SG, CDDT, MCS, VVS	No ACD, Positive for IgE	Lower legs, ankles, and dorsal feet	• Erythematous patches, lichenification, itching • Extensive ulcer, pain	Ulcer area: ∼2,000 → 0 (−100%) VCSS:18→4 (−78%)	28

*AD*, Atopic dermatitis; *ACD*, allergic contact dermatitis; *AFT*, aescuven forte tablets; *aft*, after; *AH*, antihistamine; *bef*, before; *CVI*, chronic venous insufficiency; *CDDT*, calcium dobesilate dispersible tablets; *DT*, diosmin tablets; *DU*, Doppler ultrasound; *SD*, stasis dermatitis; *IgE*, immunoglobulin E; *LEVU*, lower extremity venous ultrasound; *MCS*, medical compression stockings; *SA*, systemic antibiotics; *SG*, systemic glucocorticoids; *TC*, topical corticosteroids; *TA*, topical antibiotics; *VVS*, varicose vein surgery.

∗ Previous therapies included topical corticosteroids and other topical agents, systemic treatments such as antihistamines, systemic corticosteroids, antibiotics, and venotonic agents, as well as local wound care, compression therapy, and/or venous surgery. All patients had failed long-term standard-of-care treatment and discontinued systemic therapies prior to dupilumab initiation.

† Ulcer area was assessed by digital planimetry using pixel-based area measurement on serial clinical photographs, with baseline images serving as within-patient references for percentage change.

We further examined lesional skin of 12 patients with SD (including patient 1), 6 with AD, and 5 healthy controls (Fig 1, *B* and *C*). The positive area percentage of key markers, including CD4, IL-4, VEGFA, and SMAD3, was quantified in dermal regions. CD4 and IL-4 levels were significantly elevated in both SD and AD compared with healthy control, indicating a shared type 2–skewed immune response. VEGFA expression was also increased in SD lesions and showed a similar upward trend in AD, reflecting active pathological angiogenesis in both conditions. To our surprise, SMAD3—the central mediator of the TGF-β signaling pathway involved in fibrosis and tissue remodeling—was relatively insufficient in SD compared with AD, highlighting distinct pathogenic programs link to impaired repair signaling in SD.

CD4^+^ Th2 cells has been linked to IL-31–associated pruritus in SD, yet other mechanisms remain largely unkown.^2^ IL-4 promotes VCAM-1 expression in venous endothelium to sustain leukocyte recruitment, and under hypoxic conditions, is associated with VEGFA-mediated vascular abnormalities.^3^ Unlike AD, SD shows feature suggestive of impaired repair responses. Given the established role of TGF-β1–SMAD3 pathway in fibroblast activation, extracellular matrix deposition, and wound healing,^4^^,^^5^ SD lesions exhibited relatively reduced SMAD3 expression compared with AD, likely predisposing to chronic ulcers. We speculate that persistent venous inflammation and angiogenic imbalance may contribute to this relative suppression, although the precise mechanisms remain unclear and warrant further investigation. Dupilumab, by blocking IL-4Rα, was associated with improvement of ulcer healing in our patients. This study is limited by the small sample size and retrospective design, and the absence of paired pre-treatment and post-treatment tissue analyses, limiting mechanistic interpretation. In summary, refractory SD combines type 2 inflammation, abnormal angiogenesis, and defective repair, and dupilumab achieved improvement in both eczematous rash and ulcers.

## Conflicts of interest

None disclosed.
